# UK Biobank: a globally important resource for cancer research

**DOI:** 10.1038/s41416-022-02053-5

**Published:** 2022-11-19

**Authors:** Megan C. Conroy, Ben Lacey, Jelena Bešević, Wemimo Omiyale, Qi Feng, Mark Effingham, Jonathan Sellers, Simon Sheard, Mahesh Pancholi, Gareth Gregory, John Busby, Rory Collins, Naomi E. Allen

**Affiliations:** 1 Nuffield Department of Population Health (NDPH), University of Oxford, Oxford, UK; 2 UK Biobank, Stockport, Greater Manchester UK

**Keywords:** Epidemiology, Research data, Cancer epidemiology, Genetics research

## Abstract

UK Biobank is a large-scale prospective study with deep phenotyping and genomic data. Its open-access policy allows researchers worldwide, from academia or industry, to perform health research in the public interest. Between 2006 and 2010, the study recruited 502,000 adults aged 40–69 years from the general population of the United Kingdom. At enrolment, participants provided information on a wide range of factors, physical measurements were taken, and biological samples (blood, urine and saliva) were collected for long-term storage. Participants have now been followed up for over a decade with more than 52,000 incident cancer cases recorded. The study continues to be enhanced with repeat assessments, web-based questionnaires, multi-modal imaging, and conversion of the stored biological samples to genomic and other ‘–omic’ data. The study has already demonstrated its value in enabling research into the determinants of cancer, and future planned enhancements will make the resource even more valuable to cancer researchers. Over 26,000 researchers worldwide are currently using the data, performing a wide range of cancer research. UK Biobank is uniquely placed to transform our understanding of the causes of cancer development and progression, and drive improvements in cancer treatment and prevention over the coming decades.

## Background

Cancer is now the most common cause of death in many parts of the world, including North America, Europe, Australia, and China [1–3]. However, the major determinants of many cancers remain unclear, despite decades of biological and epidemiological research [4]. UK Biobank is a large-scale biomedical database and research resource, containing in-depth information on genetic, physiological, lifestyle, and environmental factors on half a million UK participants, with their health followed up through linkage to electronic health records. The resource is available to all bona fide researchers to perform health-related research, and its unique combination of scale, depth, maturity and accessibility has led to it becoming the world’s most important biomedical resource, offering enormous potential to improve understanding of the determinants of a wide range of cancers.

The risk of developing cancer reflects the combined effect of genetic and environmental factors, each of which may have only a modest effect on cancer risk [4–6]. As such, research on the effects of these factors requires epidemiological studies that collect detailed information on a very large number of people. However, previous epidemiological studies have typically involved collection of either a large amount of data on a small number of participants, or a small amount of data on a large number of participants. By contrast, UK Biobank contains extensive questionnaire data, physical measures and biological samples for a very large number of participants (i.e., both depth and breadth have been achieved). This was made possible by the establishment of highly efficient, purpose-designed centralised processes with detailed input from UK Biobank’s extensive academic collaborative network [7].

UK Biobank was established by the Medical Research Council (UK) (MRC), Wellcome, the UK Department of Health, and the Scottish Government in response to the challenge of understanding the determinants of common complex disease [8]. Participants have been followed up for over a decade, and there are now ~50,000 incident cancer cases. From inception, the study data was intended to be made available to academic and commercial researchers worldwide and the resource is now uniquely placed to enable major scientific discoveries into the causes, treatment and prevention of cancer and other diseases. UK Biobank now receive core funding from the MRC, Wellcome, Cancer Research UK, British Heart Foundation and the National Institute of Health and Care Research [9].

## UK Biobank: data collection and enhancements

### Recruitment and data collection

Between 2006 and 2010, about 9.2 million people aged 40–69 years, who were registered with the NHS and living within reasonable travelling distance (up to 25 miles) of one of 22 assessment centres across the UK, were invited to join UK Biobank. Overall, 502,000 adults (5.5% of those invited) were recruited [10, 11]. Participants underwent an extensive range of baseline assessments, including touchscreen questionnaires on sociodemographic factors, family history, lifestyle, medical history, cognitive function tests and environmental exposures. Physical measurements were taken, including blood pressure, bone mineral density, hand grip strength, eye and lung function, and cardiorespiratory fitness; and blood, urine and saliva samples were collected for long-term storage (Table 1) [7, 12]. A proportion of the cohort also underwent an eye examination (including refractive index, intraocular pressure, retinal photograph, and optical coherence tomography), a hearing test, a cardiorespiratory fitness test with 4 lead ECG test, calcaneal ultrasound for bone density, and pulse wave velocity of arterial stiffness.

**Table 1:** Overview of the questionnaire, physical measurement, and other data in UK Biobank.

Data type	Details	Number of participants
Touchscreen questionnaires	Sociodemographic factors, family history, psychosocial factors, environment, lifestyle, medical history	Baseline: 500,000; Resurvey: 20,000; At imaging: 100,000^†^; At repeat imaging: 70,000^†^
Physical measures	Blood pressure, hand grip strength, anthropometry, spirometry, heel bone density, cognitive function, arterial stiffness,^*^ hearing,^*^ eye exam,^*^ fitness test (ECG at rest and during exercise)^*^	
Web-based questionnaires	24-h diet ×4 occasions	210,000
	Cognitive function	125,000
	Occupational history	121,500
	Food preferences	182,000
	Mental health	157,000
	Digestive health	175,000
	Pain	167,000
Environmental linkages	Air and noise pollution. Built environment. Greenspace and coastal proximity. Air temperature. Road network attributes	Baseline: 500,000
Physical activity monitoring	Accelerometry data on duration and intensity of physical activity	100,000 Repeat: 2500 ×4
Imaging assessment	Brain, cardiac, full-body MRI; full-body DEXA; carotid ultrasound, 12-lead ECG	100,000 first imaging visit^†^ 70,000 repeat imaging visit^†^
Cardiac monitor	14 days of continual ECG	35,000 (pending)

^*^Available in a subset of the cohort at baseline (arterial stiffness and hearing data collected for 170,000 participants at recruitment; eye examination and fitness data collected for 100,000 participants at recruitment).

^†^Imaging currently available in 50,000 participants, and 5000 repeat images.

The original sample size was selected to maximise the number of incident cases of a wide range of important diseases to support the reliable investigation of their potential determinants. Power calculations prior to study recruitment indicated that for an exposure in 10% of the cohort, 5000 cases of a health outcome (i.e., 1% of the cohort) would be required to identify a minimum detectable odds ratio of 1.26 at a critical *P* value of 10^−4^ [8]. For gene-by-environment analyses, assuming a 10% prevalence of both the genotype and environmental exposure, 5000 cases would enable the identification of a minimum odds ratio of 1.98. As a result, very large numbers of participants are needed to identify large numbers of cases of particular diseases during a reasonable follow-up period. However, despite the large sample size of UK Biobank, some gene-by-environment analyses will not be possible for rare exposures or outcomes, and pooling data across other cohort studies is necessary to ensure adequate sample sizes for reliable investigation.

Due to the volunteer nature of the cohort, the UK Biobank cohort is not representative of the current general UK population in a number of ways [11]. However, the extent to which this actually matters depends on the aims of the specific research question. To ensure associations are generalisable to a wider population (or future populations), what may be more important is to have sufficiently large numbers of participants with different levels of exposures and incident disease [13]. For example, although the UK Biobank cohort contains a lower proportion of participants who live in more deprived areas compared with the UK population (16% [82,000] vs. 33% in the UK population), it still includes sufficiently large numbers to allow associations of socio-economic deprivation with disease risk to be investigated with high internal validity.

As a consequence of the healthy volunteer effect, cancer incidence rates are generally lower in UK Biobank in comparison to the general UK population but this varies by cancer site, as previously reported [11]. As such, UK Biobank should not be used to estimate cancer prevalence or incidence rates, but can be used to assess reliably the aetiological associations between exposures and cancer outcomes.

### Outcome ascertainment

**Fig. 1:**
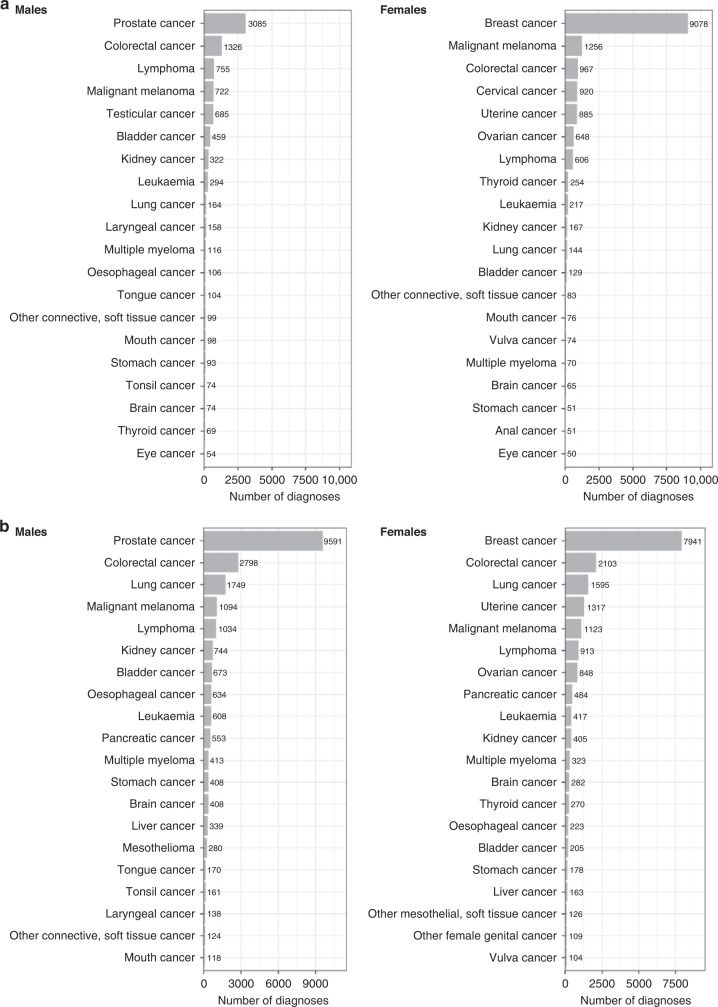
Number of prevalent and incident cancer in UK Biobank. **a** Prevalent cancers by sex; **b** incident cancers by sex. Cancer registry data available until February 29, 2020 for England and Wales and January 31, 2021 for Scotland. Graphs reproduced from UK Biobank cancer summary report (https://biobank.ndph.ox.ac.uk/~bbdatan/CancerSummaryReport.html accessed 27/9/2022).

Participants provided consent for UK Biobank to follow their health over time through linkage to electronic medical and other health-related records. To date, linkage has been achieved to national death and cancer registries and hospital inpatient admissions (including critical care), with linkage to primary care available for ~45% of the cohort (Table 2 and Fig. 1). Cancer registry data provides curated data on the histological tumour type and date of diagnosis, both prior to recruitment (with data from the mid-1950s onwards) and during follow-up. Cancer registry data are considered the gold standard method for ascertaining cancer outcomes in the UK, owing to mandatory reporting of cancer outcomes within the NHS [14]. However, due to data being curated from multiple sources, there is a time lag to completeness, with data from cancer registries usually complete within 2 years of diagnosis. Primary care records include data on rapid referral under the 2-week pathway, cancer-relevant biomarkers (e.g., prostate-specific antigen testing and CA-125 measurements) and other information on the route to diagnosis, co-morbidities and medication use. Linked health data are updated approximately annually within UK Biobank (except GP data). UK Biobank also periodically contacts participants directly to obtain information on health-related conditions that are not well-captured in healthcare records (e.g., cognitive function, mental health, pain, etc.) through a series of web-based questionnaires (Table 1). These data are potentially important for cancer research as they can be used, for example, to assess pain among cancer patients, as well as enabling research into cognition and mental health of cancer survivors. Further details on data linkages, cleaning, validation and data availability (including summary statistics for all data fields) can be found on the UK Biobank data showcase webpage (https://biobank.ctsu.ox.ac.uk/crystal/).

**Table 2:** Health-related linkages in UK Biobank.

Data type	Details	Number of participants	Date coverage
Death registry	Date and cause of death from national registries	Whole cohort	England & Wales: 2006–2021; Scotland: 2006–2021
Cancer registry	Date of diagnosis and type of cancer from national registries		England & Wales: 1971–2016; Scotland: 1957–2015
Hospital admissions	Date of admission and discharge, diagnoses, procedures from inpatient records, including critical care		England: 1997–2020; Wales: 1999–2018; Scotland: 1981–2020
Primary care	Date of appointment, diagnoses, symptoms, prescriptions, referrals, consultations	230,000	England: 1938–2016; Wales: 1948–2017; Scotland: 1939–2017

### Enhancements to the resource

Following the baseline assessment between 2006 and 2010, additional data have continued to be collected to enhance the value of the resource for health-related research. During 2013, a reasonably representative sample of about 20,000 participants was invited back for a repeat of the baseline assessment visit (including sample collection and storage) in order that researchers can make essential allowance for regression dilution bias due to measurement error and within-person fluctuations in exposure levels in their disease association analyses [15].

UK Biobank has also collected data on physical activity using wrist-worn accelerometers in 100,000 participants between 2013 and 2015, which was repeated on a seasonal basis in a subset of 2500 participants a few years later to assess changes in activity over time [16]. National guidelines on physical activity are based mainly on epidemiological studies that have used self-reported data, and the accelerometer data [16] in UK Biobank is now enabling robust research into the associations of objectively measured physical activity and sleep patterns with health outcomes (Table 1).

In 2014, UK Biobank initiated the world’s largest imaging sub-study, which aims to recruit up to 100,000 participants to undergo magnetic resonance imaging (MRI) of the brain, heart, and body, whole-body dual-energy X-ray absorptiometry (DXA), carotid ultrasound, together with a repeat of the baseline assessment, including questionnaires, physical measures and biological sampling (blood and urine) [17]. By the end of 2021, 50,000 participants had been scanned at one of four bespoke UK Biobank imaging centres, with a subset also invited to wear a cardiac monitor for 2 weeks. Furthermore, repeat imaging of up to 60,000 participants has also started, allowing research into the relationship between changes in internal physiology (such as muscle and fat distribution), and risk of disease onset and progression, which is likely to be of particular value for research into identifying early detection biomarkers and for refining risk prediction models.

Plans are underway to incorporate further information on cancer phenotyping by expanding its linkage to national datasets with information on tumour aggressiveness (i.e., stage, grade), morphology, and treatment (including radiotherapy, chemotherapy, immunotherapy and hormone treatments). These data will allow for more detailed research into risk factors for different cancer subtypes, as well as identifying suitable prognostic markers for survival and provide data for pharmacogenomics research [18]. However, researchers should be aware that data completeness varies by cancer site, as these data are not compulsory to provide to the National Cancer Registration and Analysis service [19]. For tumour grade, the amount of missing data varies from 0% to about 70%, with breast, colorectal, pancreatic and oesophageal cancers having the most complete data, and brain and uterine having the least. Tumour stage data are available for between 55 and 90% of cancers, with brain, hepatobiliary and pancreatic being most complete and colorectal, endometrial and ovarian the least. Pilot studies are also currently underway to assess the feasibility of incorporating digitised histopathology slides into UK Biobank to enable researchers to ascertain different morphological subtypes of cancer. It may also be possible to link to datasets that contain information on the molecular characterisation of cancer subtypes (e.g., biomarkers or genetic changes in cancer tissue), which will accelerate research into their aetiological pathways and how best to treat and manage them. For example, it is now well-established that colorectal cancer evolves through multiple pathways, which can be classified according to their molecular features (e.g., DNA microsatellite instability and methylation) [20]. Detailed phenotyping of cancers, together with better characterisation of key exposures (such as imaging-derived body composition and genomic data), will support powerful research into the determinants of different cancer subtypes.

UK Biobank’s policy has been, wherever possible, to perform cohort-wide assays on the biological samples, thereby allowing the limited biological samples to be used for the widest possible range of research [21]. This unique approach facilitates good quality control and effective management of the limited and depletable sample volume [21]. The availability of a wide range of biomarkers in all 500,000 participants increases the resource’s utility, as it allows research between biomarkers and a wide range of outcomes (which is simply not possible if using a case–control design). To date, cohort-wide data have been made available on: haematological and biochemistry assays [22] (including several biomarkers of relevance to cancer research, such as sex hormones and insulin-like growth factor-I); leukocyte telomere length; [23] and genome-wide genotyping using an Affymetrix array of ~850,000 variants, with imputation on >90 million variants (Table 3) [24]. In addition, industry consortia have performed whole-exome [25] and whole-genome sequencing for all 500,000 participants, making this biomedical database the world’s largest resource for scientists to gain valuable insights into the genetic determinants of disease. Of course, the availability of genetic data - coupled with lifestyle information and clinical outcomes on such a large-scale - will also accelerate the identification of potential drug targets.

**Table 3:** Assay data generated from biological samples in UK Biobank.

Sample assay	Details	Number of participants
Biochemistry markers	Biomarkers assayed in plasma, serum, red blood cells, and urine samples; includes established risk factors for disease (e.g., lipids for vascular disease, sex hormones for cancer), diagnostic measures (e.g., HbA1c for diabetes and rheumatoid factor for arthritis), and other measures (such as liver and renal function tests)	Baseline: 500,000; Resurvey: 20,000
Infectious agents	20 major pathogens (including herpes viruses, hepatitis B and C viruses, HIV, HPV, *C. trachomatis, H. pylori*)	Baseline: 50,000*
Genotyping	Genome-wide genotyping chip with >800,000 markers; imputed to ~96 M variants using the Haplotype Reference Consortium and UK10K haplotype reference panels	Baseline: 488,000
Whole-Exome Sequencing	Whole-exome sequencing data (covers 1–2% of the genome)—multi-sample joint call data, variant-level and raw sequence data	Baseline: 488,000
Whole-Genome Sequencing	Whole-genome sequencing data (full genome)—multi-sample joint call data, variant-level and raw sequence data	Baseline: 488,000^†^
Telomeres	Telomere length	Baseline: 500,000; Resurvey: 20,000
NMR metabolomics	Approx. 200 circulating metabolites, predominantly lipids	Baseline: 120,000; Resurvey: 3000
Proteomics	Approx. 3000 circulating proteins	Baseline: 50,000 (pending^‡^)

^*^Currently available for 10,000 participants.

^†^Currently available for 200,000 participants, full cohort expected to be available in 2023.

^‡^Data expected to be available in 2022.

Arising from previous consortia to fund genetic sequencing, a pharmaceutical consortium is investing in proteomic measurements for 3000 proteins in 57,000 participants using the O-LINK platform. These samples were selected randomly (~45,000) or enhanced for diseases of interest by the consortium members (~8000). These data are expected to be released in 2023 (Table 3), and there is significant interest in extending these measures to the full cohort to accelerate the development of drug targets and identifying early detection biomarkers [26].

Metabolomics assays using nuclear magnetic resonance (NMR) spectroscopy (funded by Nightingale Health) are underway for all 500,000 participants, with the first tranche of data released in 2021 for >200 circulating metabolites for 120,000 participants at baseline and 3000 participants at resurvey [27]. Data on serological markers of infectious agents, including a number of known or potential oncogenic pathogens, are also available for a subset of participants [28], with recent funding (from Open Philanthropy) to extend these data to an additional 50,000 participants (Table 3).

Dates for the future release of data, such as the enhanced cancer data and proteomics, are available on our website (https://www.ukbiobank.ac.uk/enable-your-research/about-our-data/future-data-release-timelines?src=future_timelines).

UK Biobank works with, and is guided by, the research community to ensure the resource is continually enhanced, and welcomes proposals from researchers to improve its utility. In addition to samples being available for assay, proposals for exposure and outcome measurement to develop the resource are considered. Researchers that wish to discuss potential enhancements (such as further linkages) that would be beneficial to the cancer research community are encouraged to contact UK Biobank’s access team (https://www.ukbiobank.ac.uk/learn-more-about-uk-biobank/contact-us).

## UK Biobank and cancer research

UK Biobank is an important resource for population-based cancer research. There are already over 43,000 incident cancer cases recorded to date in the national cancer registry among UK Biobank participants (in addition to the 26,000 prevalent cases at baseline, including 9000 prevalent breast cancers, 3000 prevalent prostate cancers and 2200 prevalent colorectal cancers (Fig. 1)). This includes 9500 incident prostate cancers, 7900 incident breast cancers, 4900 incident colorectal cancers and 3300 incident lung cancers (Fig. 1). Even for some relatively rare cancers, such as renal cell carcinoma and endometrial cancer, there are already over 1100 incident cases. As the cohort ages (the average age is now 70 years), the number of cancer cases will increase substantially, with incident prostate, breast, colorectal and lung cancers predicted to increase to 16,000, 14,000, 8000 and 6000 cases, respectively, by 2027 (these estimated numbers are adjusted for age, sex, and the healthy volunteer effect seen in UK Biobank). The full cancer reports and methodology can be accessed on the UK Biobank data website [29, 30].

UK Biobank is particularly suited to enable studies on the determinants of disease; identifying risk factors that make people more or less likely to develop a particular disease, and quantifying the strength of the associations. This can often be challenging using small-scale studies, due to the limited power from low numbers of disease events. UK Biobank’s size, together with its deep phenotyping, allow associations to be quantified with greater precision, and across levels of other risk factors. Furthermore, variation in the strength of the associations across a broad range of demographic, socio-economic, and lifestyle characteristics can be used to assess the generalisability of the associations to important population subgroups [11, 31].

Since the release of genome-wide genotyping data for all UK Biobank participants in 2017, the study has played a central role in accelerating the identification of genetic variants associated with cancer risk. Recent studies using UK Biobank data have identified new susceptibility loci for specific cancers, including endometrial cancer [32], colon cancer [33] and cervical cancer [34]. Such studies are particularly valuable in understanding the biological mechanisms underlying the development of cancer. For example, many genetic variants associated with cervical cancer risk are in the region of *PAX8, CLPTM1L* and *HLA* genes, suggesting a disruption in apoptotic and immune function pathways [34]. Research has also identified genes that affect the risk of more than one type of cancer (many of which appear to be regulatory elements and/or influence cross-tissue gene expression), offering further insight into the complex genetic architecture of cross-cancer susceptibility [35]. Further, research has identified shared genetics between known cancer risk factors and cancer development (such as alcohol consumption and oral cancer [36] and obesity and progression of a number of cancers [37]) which will help to disentangle the causal pathways of known associations.

Genotyping data have also facilitated causal inference through the use of Mendelian randomisation, a technique whereby genetic variants that are associated with a given exposure are used to investigate the causality of associations between an exposure and outcome of interest [38]. Mendelian randomisation takes advantage of the random assortment of genes from parents to offspring that occurs during gamete formation and conception to mimic the effect of a randomised controlled trial for a particular exposure. Analyses using Mendelian randomisation have supported the causality of the associations of circulating insulin-like growth factor-1 concentration with colorectal, breast and prostate cancer risk [39–42], obesity with endometrial cancer [43], and height with overall cancer risk [44]; but refute previous observational evidence for an inverse association between vitamin D concentration and colorectal cancer risk [45].

In addition to research on the causes of disease, GWAS data can also be used to construct polygenic risk scores, which combine the effects of genetic variants (each of which may have only a small effect on cancer risk) to improve risk prediction [46–51]. These scores could then be used to stratify the population according to their genetic risk, or used to enhance existing risk prediction algorithms (such as QCancer for cancer risk [52, 53]) that use information on sociodemographic, lifestyle or clinical factors. Polygenic risk scores have been developed for a wide range of cancers using UK Biobank data. For example, an academic consortia (Breast Cancer Association Consortium) have developed a polygenic risk score for breast cancer composed of 313 genetic variants, with those in the highest group having a lifetime risk of about 30% for developing ER + breast cancer, compared to 2% in the lowest, with a range of 0.55–4% for ER− disease [54]. Many of those in the high PRS category do not have a strong family history of breast cancer, so would not be identified by standard risk screening tools. The clinical utility of polygenic risk scores is being assessed, but such scores may well be used to inform clinical decision-making or to inform screening programmes (e.g., to target individuals with a high polygenic risk score for certain cancers to undergo earlier or higher frequency screening programmes [54, 55]).

The release of whole-exome and whole-genome sequencing for 500,000 participants will be extremely valuable to research into new cancer therapies. In particular, variants in the exome region of the genome (which encode for proteins) can be used to identify genetic variants of particular relevance for drug discovery. For example, a study using UK Biobank data has already found that a genetic variant in the gene that encodes the *GPR75* receptor is associated with a significantly lower rate of obesity in homozygous carriers [56]. This has subsequently been confirmed in animal models [56], and paves the way for pharmaceutical trials to develop new treatments for obesity. Whole-genome sequencing makes it possible for scientists to investigate the impact of coding and non-coding DNA and of repeated, missing or extra sequences of DNA, on disease risk. These data offer an opportunity to understand the potential impact of inhibiting or agonising the product of a gene, with relevance to drug development [57]. It also allows the detection of rare, non-coding variants that will help us understand gene regulation and disease mechanisms, as well as the identification of structural variations, such as short tandem repeats, which can be used to further understand the aetiologies of complex diseases.

In addition to understanding the genetic determinants of disease, the rich characterisation of participants in UK Biobank has been used to assess the behavioural and environmental determinants of cancer, such as from dietary factors [58, 59] and physical activity [60–62] to shift work [63] and exogenous hormone use [64, 65], with some results directly impacting public health policy. For example, researchers using dietary data collected at baseline and at resurvey (supplemented by the dietary web-based questionnaire) found higher consumption of red and processed meat was positively related to risk of colorectal rectal, even within the current UK guidelines that recommend no more than 90 g of red and processed meat per day [59]. Researchers have also used accelerometer-based measures of physical activity to improve understanding of the associations of physical activity and risk of breast cancer [66]. The study found that greater physical activity was associated with a reduction in breast cancer risk in both pre- and post-menopausal women, independent of any association it may have on risk through its effects on adiposity.

A wide range of anthropometric measures were collected as part of the baseline survey in UK Biobank, and these have been used to assess the impact of adiposity on cancer risk. A recent study assessed the association of six adiposity-related markers (including body-mass index, body fat percentage, waist-hip ratio, waist-height ratio, and waist and hip circumference), with risk of 24 different cancers [67]. The study found strong associations with a number of cancers, including cancers of the stomach cardia, gallbladder, liver and kidney. The availability of imaging data on large numbers of participants will substantially enhance research into the effect of adiposity (and other endogenous markers of body size and structure and composition) with disease risk, allowing more precise analyses of the risks associated with specific measures of body composition. Imaging-derived adiposity measures from DXA and MRI images have already been used to assess the relation between the distribution of body fat and risk of several cancers. For example, a recent study found that for a given level of total body fat, increased central adiposity was associated with an increased risk of colorectal cancer, but increased hip fat was associated with a reduced colorectal cancer risk [68].

The cohort-wide assays performed on the blood samples from all 500,000 participants are already enabling robust research into the role of sex hormones and risk of cancer onset and progression. For example, analyses using UK Biobank have shown that the risk of endometrial cancer is positively related to circulating levels of total and free testosterone but inversely related to levels of sex hormone-binding globulin [69] with Mendelian randomisation analyses supporting the causality of these association [69]. In men, higher free testosterone, but not total testosterone, has been found to be associated with risk of prostate cancer [41]. Conversely, biomarkers of inflammation do not appear to be related to risk of glioma [70] and circulating lipid levels are not strongly associated with ovarian cancer risk [71]. Biomarkers can also be used to investigate the pathways between known risk factors and cancer diagnosis, with research showing that the increased risk of colorectal cancer associated with obesity is unlikely to be driven by adiposity-induced chronic inflammation, insulin resistance or sex-steroid hormone levels [72]. Proteomic data, in particular, may help identify individuals at high risk of specific cancers or may aid in their diagnosis, with small-scale analyses in other cohorts indicating its utility [73]. Proteomics—particularly when combined with genetics and metabolomics data in a single cohort—will enhance the opportunities to investigate the biological pathways by which genes affect cancer risk, with the potential to identify novel drug targets and treatments [73].

With such a complex dataset, researchers have employed artificial intelligence tools to identify risk factors for cancer incidence and to improve risk prediction models for cancer onset and survival [74, 75]. For example, machine-learning algorithms have been used to predict overall survival in breast cancer patients from whole-exome sequencing data in UK Biobank [76]. Researchers have also used machine learning to derive phenotypes from complex data, such as sleep phenotypes from the accelerometer data or imaging phenotypes, such as organ segmentation, from the MRI data [77–79]. Machine-learning methods allow the relationships among different variables and types of data to be learnt from the data itself, and this may have advantages over classical statistical methods, where the relationships among variables need to be pre-specified, and only a limited number of factors can be modelled at a time [80].

## Accessing UK Biobank data

**Fig. 2:**
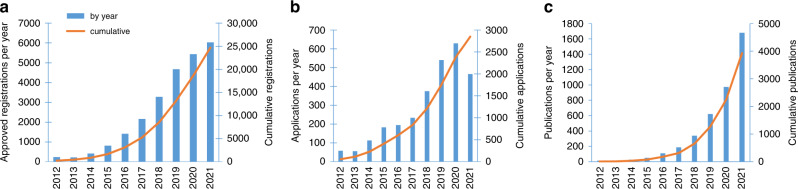
UK Biobank registrations, applications and publications. **a** Researchers registrations; **b** Project applications; **c** Publications, by year.

What makes UK Biobank so unique is the easy accessibility of a vast range of data on 500,000 participants to all bona fide academic or commercial researchers, anywhere in the world [81]. Researchers must register prior to submitting an application, and the application must be for health-related research that is in the public interest. UK Biobank has a policy of no preferential access, ensuring all applicants (whether academic, governmental, charitable or commercial) are treated in the same way [82], and has seen an exponential rise in registered researchers, with over 25,000 registered researchers and 2800 applications by the end of 2021 (Fig. 2). This has been borne out with over 1600 publications arising from UKB data in 2021 alone (Fig. 2).

UK Biobank is a registered charity, and manages access fees on a cost recovery basis, which are reviewed on a periodic basis and are subsidised for student projects and research groups based in low and low-to-middle-income countries [83]. Applications to access biological samples are reviewed more stringently, due to the limited and depletable nature of the samples. Information on how to access the dataset can be found on the UK Biobank website (https://www.ukbiobank.ac.uk/enable-your-research/apply-for-access).

The data included in the UK Biobank resource is expected to grow to 50 petabytes by 2027. In the past, UK Biobank data have been provided to approved researchers for download, which requires a non-trivial level of local computing power and storage. The continuing expansion of data requires a more democratic approach to ensure the data are available to all researchers. Consequently, UK Biobank has made available a new cloud-based Research Analysis Platform, developed by DNAnexus (Mountain View, CA) and hosted by Amazon Web Services (London, UK). This ensures that access to UK Biobank data will remain open to all, and not just those with the information technology infrastructure to store and analyse such large data. Further, research credits to subsidise the cost of running analyses on the Research Analysis Platform have been provided to support early career researchers and those from low-and middle-income countries (https://www.ukbiobank.ac.uk/enable-your-research/research-analysis-platform/the-uk-biobank-platform-credits-programme).

## Conclusion

UK Biobank is a large-scale prospective study with deep phenotyping and genomic data. Easy accessibility to this vast biomedical resource allows researchers from around the world to make scientific discoveries to improve population health. The sheer depth and breadth of data mean that UK Biobank is now arguably the world’s most important health resource for understanding the determinants major diseases in middle and old age; it is now being used by over 25,000 researchers internationally and generating thousands of peer-reviewed publications. The resource has already demonstrated it value in enabling novel and robust research into the determinants of cancer, and will only grow in value as more incident cancer cases occur over time. In particular, the combination of whole-genome sequencing, imaging, proteomics, and metabolomic data, will enable the world’s best minds to transform our understanding of the causes of cancer development and progression and drive improvements in cancer treatment and prevention.

## Data Availability

UK Biobank is an open-access resource. Applications to access the data from bone fide researchers can be made at https://www.ukbiobank.ac.uk/enable-your-research/apply-for-access.
